# Supplementary material for: Occult polyclonality of preclinical pancreatic cancer models drives in vitro evolution
Source: Nat Commun. 2022 Jun 25;13:3652. doi: 10.1038/s41467-022-31376-3 (PMC9233687; doi:10.1038/s41467-022-31376-3)

Supplementary Fig. 1) Phenotype mapping and cladogram analysis of cell lines to measure evolutionary relatedness based on scRNAseq data. a) Phenotype mapping as a function of relative diffusion space (indicated by heatmap legend) of all cell lines. **b)** Cladogram depiction of diffusion space between cell lines. The x-axis indicates the sample names and y-axis indicates the phenotypic distances.

a)

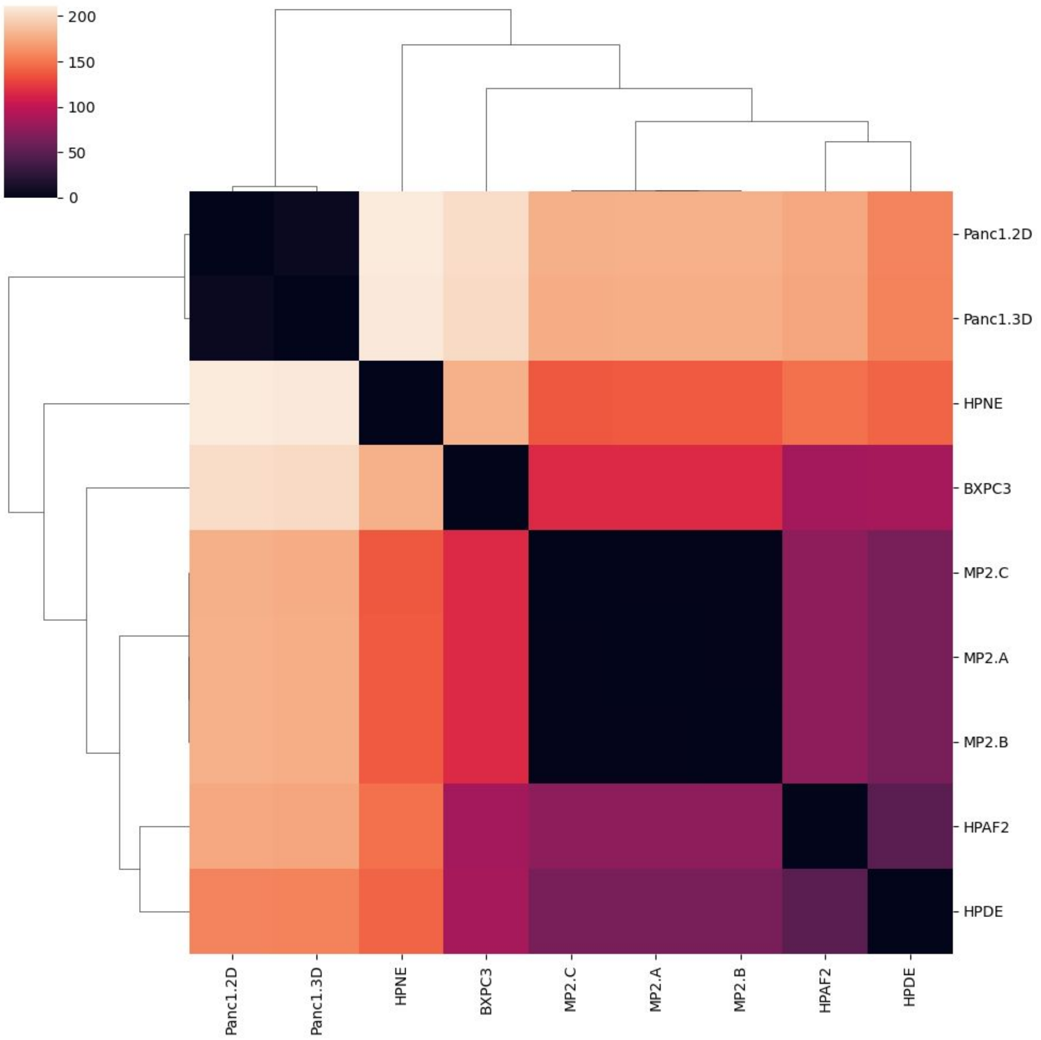

b)

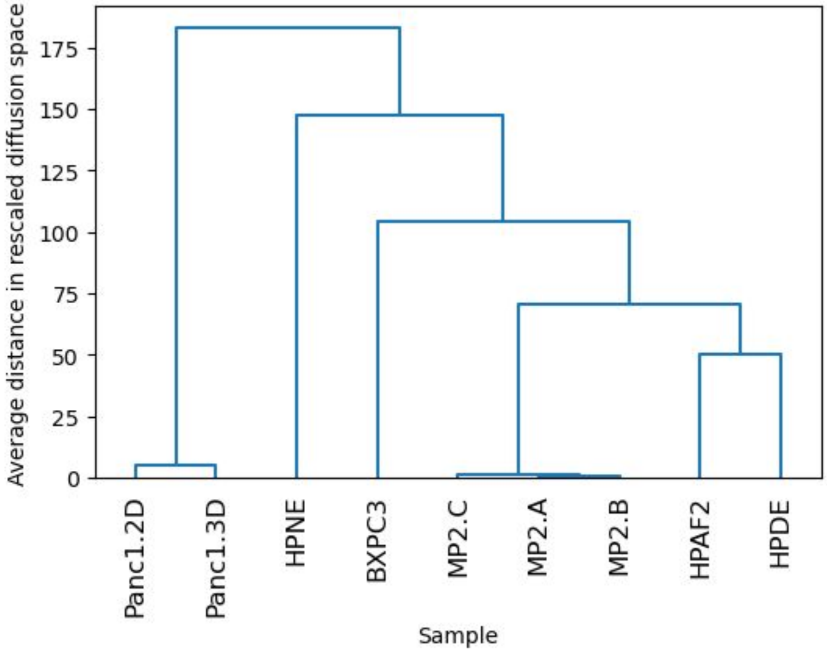

Supplementary Fig. 2) scCNV profiles of PDAC cell lines reveal subclonal heterogeneity. For all samples depicted here (**a-c**), columns indicate chromosomes (numbers labeled in gray and white), rows indicate individual cells organized into clonal clades. Phylogeny determined using 10x Genomics scCNV analysis software.

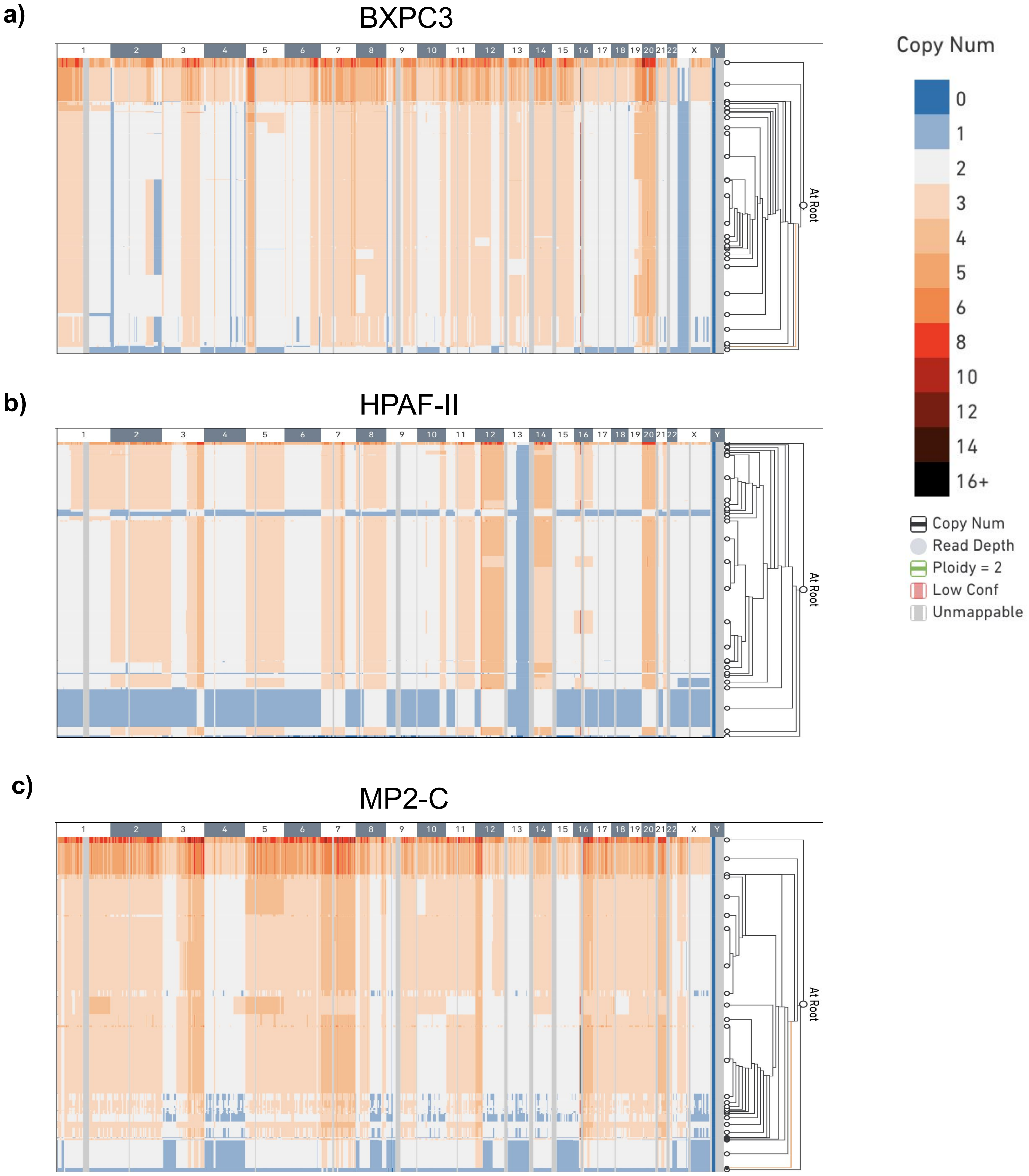

Supplementary Fig. 3) a) Segmentation plotting derived from normalized copy number ratios of scCNV clones from each cell line. Columns indicate chromosomes, rows indicate ploidy ratio.

a)

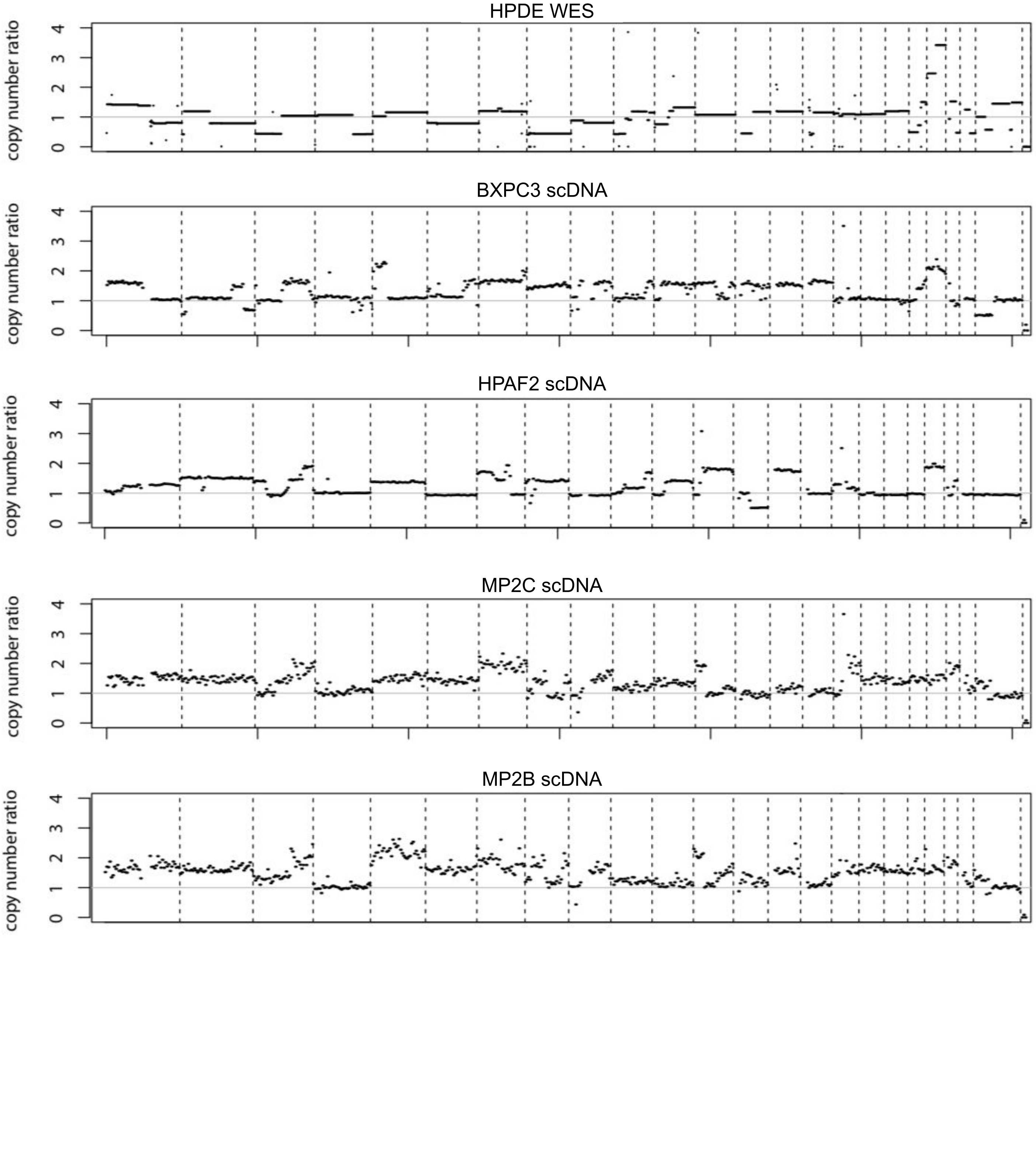

Supplementary Fig. 4) PDAC cell lines harbor differential CNV events at oncogenic loci. Heatmap with copy number per cell (rows) per gene (columns) for a series of known oncogenes, for **(a)** HPNE cells and **(b)** all non-HPNE cell lines with scCNV profiled. Showing a random subset (1000) of cells per cell line.

a)

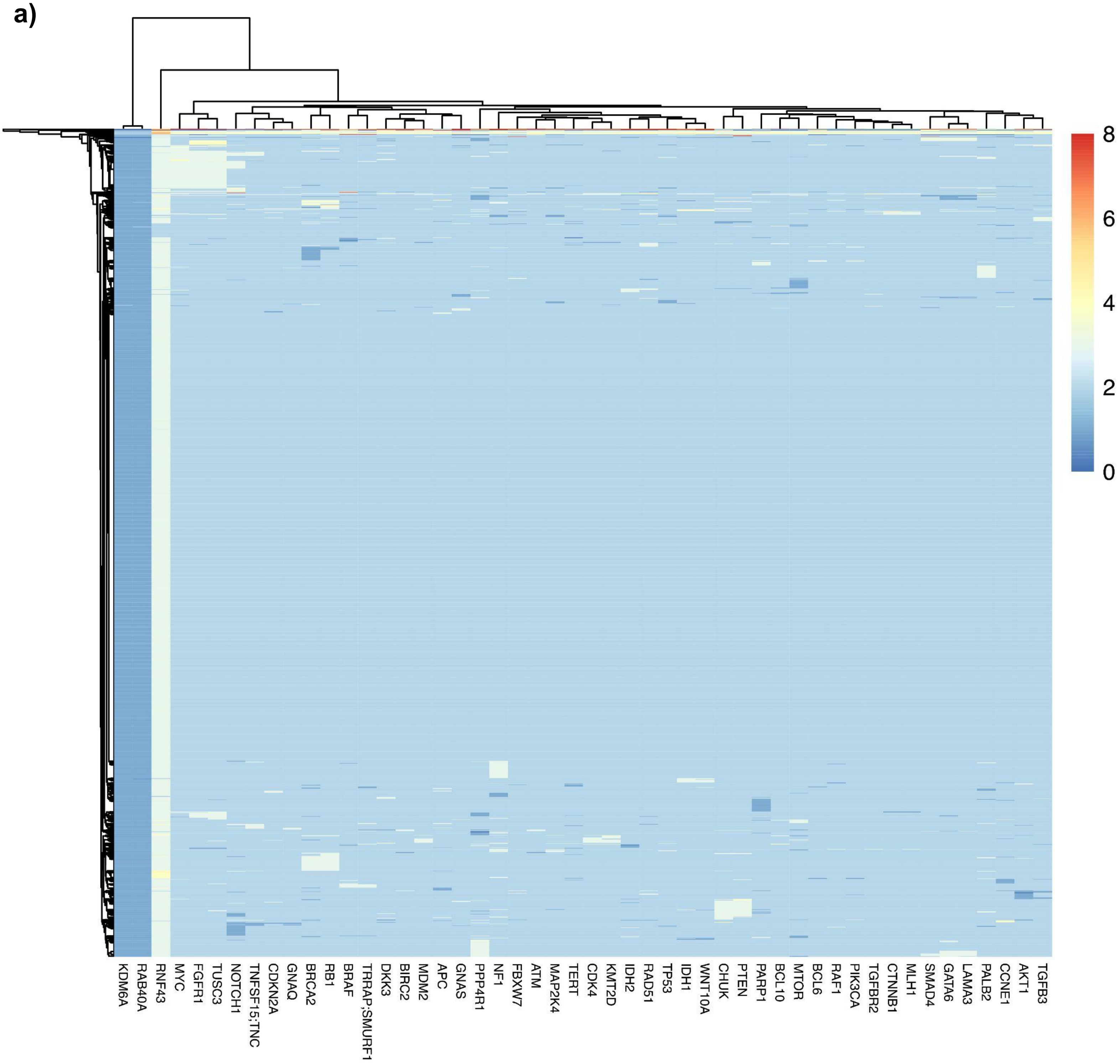

Supplementary Fig. 4, continued.

b)

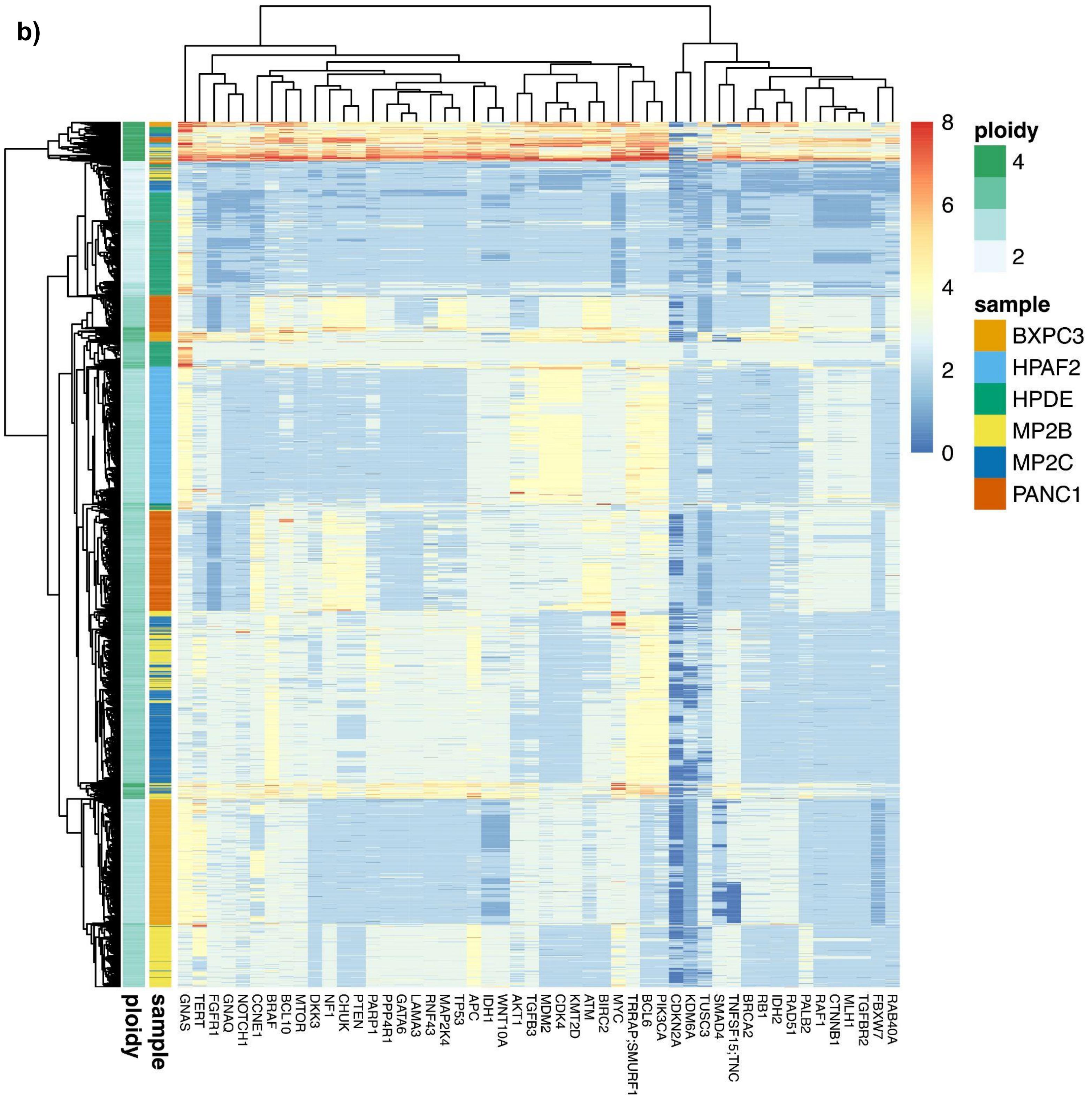

Supplementary Fig. 5) FISH validation of select scCNV amplifications.
a) FISH validation of amplifications of AURKA (green) and TERT (red) in HPNE, HPAF-II, BxPC3 and HPDE cell lines, corroborating reported scCNV findings of amplification events at these loci. Two-tailed P values determined using Mann Whitney tests for pairwise comparisons in Prism 9 (v9.0.0). For TERT: HPNE-HPDE pval < 0.0001, HPAF2-HPNE pval = 0.4076, BXPC3-HPNE pval < 0.0001. For AURKA: HPNE-HPDE pval < 0.0001, HPAF2-HPNE pval < 0.0001, BXPC3-HPNE pval < 0.0001. Scale bar = 5 microns.

a)

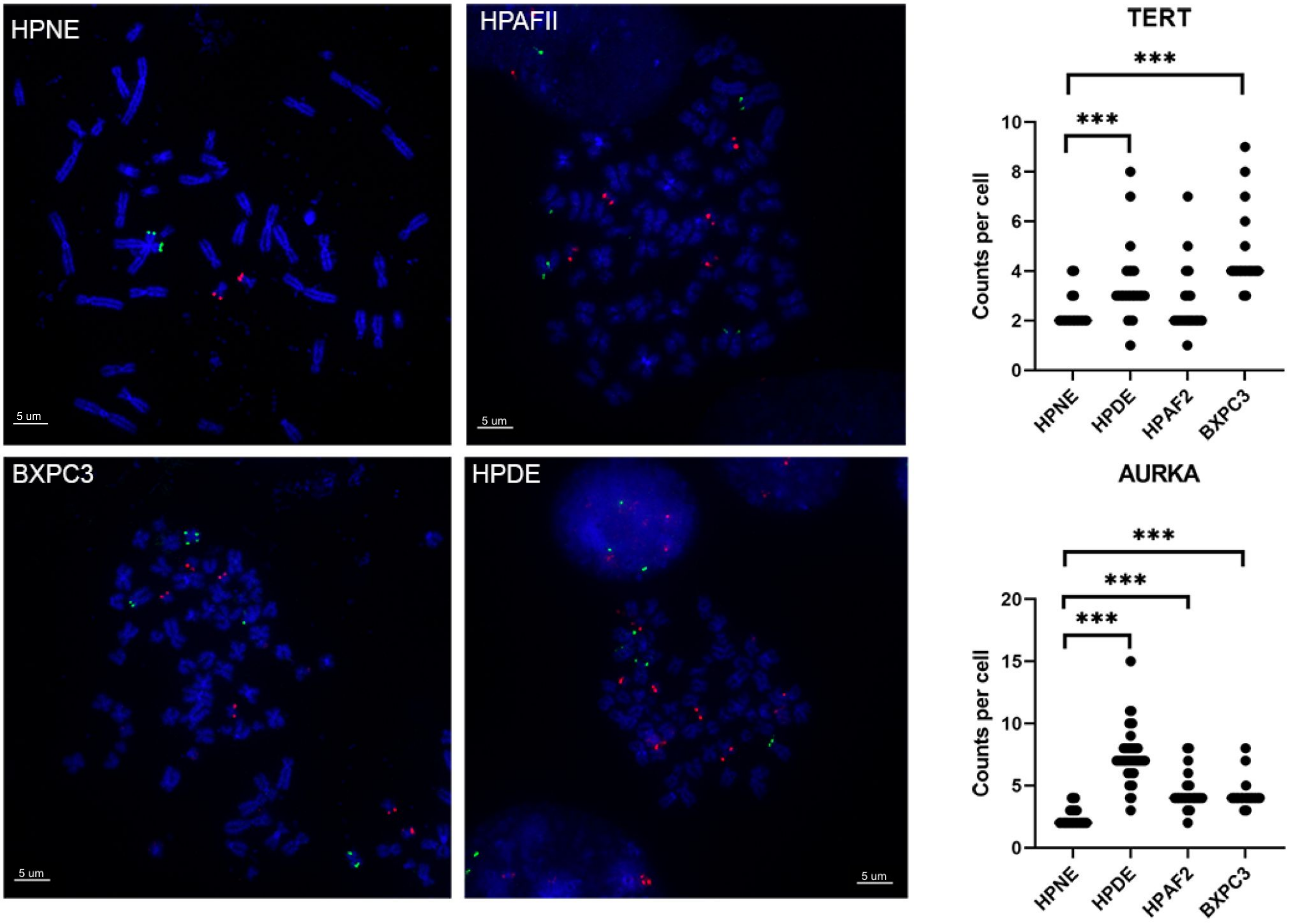

Supplementary Fig. 6) Hotspot KRAS mutations confirmed in PDAC cell lines. Droplet Digital PCR (ddPCR) results of KRAS probes for hotspot mutations G12C, G12D, and G12V applied to MP2 samples A-C and Panc1. **a-c)** Confirms same KRAS G12C mutation in all 3 cell lines. **d)** Panc1 ddPCR results indicating both KRAS G12D mutation.

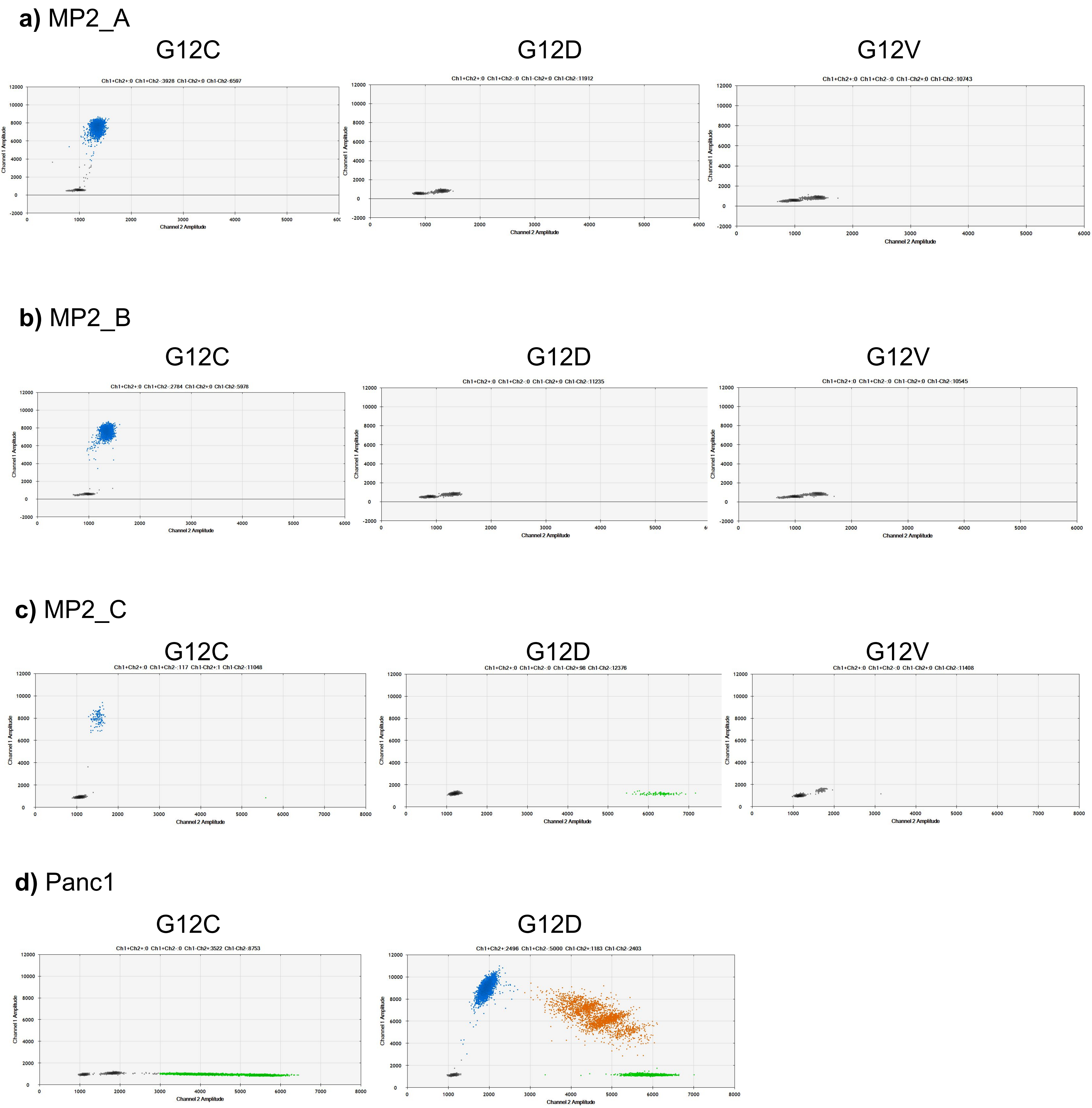

Supplementary Table 1. Summary of GSEA results on gene lists derived from scCNV clones, which were analyzed with clonealign and subsequently mapped so scRNAseq data. CNV_events describe the major amplification or deletion events that were common to groups of cells per sample, and therefore used to define clonal families by clonealign. Cell_count depicts the number of cells harboring a set of CNV events. Hallmark_enriched and Oncogenic_enriched list gene set pathways that were enriched in scRNAseq clusters corresponding to scCNV-defined clones.

**CNV_clone**	**CNV_events**	**cell_count**	**Hallmark_enriched**	**Oncogenic_enriched**
**MP2_B_C1**	chr7_p.amp, chr10_p.del, chr10_q.del, chr12_q_subseg.neu	1096	MYC, Glycolysis, Oxidative Phosphorylation, E2F	LEF1, P53, Cyclin D1
**MP2_B_C2**	chr7_p.neu,chr10_p.neu,chr10_q.neu,chr12_q_subseg.del	813	TNFA, UV Response, Apical Junction, KRAS, MTORC1, Unfolded Protein Response	MTOR, EIF4E, RAF, NEF2L2
**MP2_C_C1 + MP2_C_C3**	chr5.amp, chr10_p.del, chr10_q.neu, chr14_q.neu; chr5.neu,chr10_p.del,chr10_q.del,chr14_q.del	1208	Angiogenesis, P53	CRX, CAHOY Astroglial, ESC_V6.5, VEGF
**MP2_C_C2**	chr5.neu, chr10.neu,chr14.neu	871	N/A	N/A
**MP2_C_C3**	chr5.neu,chr10_p.del,chr10_q.del,chr14_q.del		combined with clone 1 as described in Main Text	N/A
**Panc1_3D_C1**	chr14_q.neu, chr15_q.del	904	TNFA, Estrogen Response Late + Early, Fatty Acid Met., Cholesterol Homeostasis, Coagulation, IFGamma Response, Inflammatory Response, IFAlpha Response	EGFR_UP.V1, PRC1_BMI_UP.V1, ERBB2_UP.V1, BCAT.100_UP.V1, PRC2_SUZ12_UP.V1, MEK_UP.V1, ESC_V6.5_UP_LATE, MEL18_DN.V1, CSR_EARLY_UP.V1, AKT_UP_MTOR_DN.V1, JNK_DN.V1, KRAS.DF.V1, ATF2_S_UP.V1_DN
**Panc1_3D_C2**	chr14_q.del, chr15_q.neu	223	N/A	EIF4E
**Panc1_2D_C1**	chr14_q.neu, chr15_q.del	3251	G2M, E2F Targets, Estrogen Response Late, Bile Acid Metabolism, Apoptosis, Cholesterol Homeostasis	N/A
**Panc1_2D_C2**	chr14_q.del, chr15_q.neu	404	N/A	N/A

Supplementary Table 2. Per reviewer request, list of Basal and Classical genes from the Moffitt Classification schema, used in this study for scRNAseq cell stratification in cell lines and PDOs.

**Moffitt Basal**	**Moffitt Classical**
AREG	AGR2
CST6	AGR3
DHRS9	ANXA10
FAM83A	BTNL8
FGFBP1	CDH17
GPR87	CEACAM6
KRT15	CLRN3
KRT17	CTSE
KRT6A	CYP3A7
KRT6C	FAM3D
KRT7	KRT20
LEMD1	LGALS4
LY6D	LYZ
S100A2	MYO1A
SCEL	PLA2G10
SERPINB3	REG4
SERPINB4	SPINK4
SLC2A1	ST6GALNAC1
SPRR1B	TFF1
SPRR3	TFF2
TNS4	TFF3
UCA1	TSPAN8
VGLL1	VSIG2

Supplementary Fig. 7) Expression of few genes dictates transcriptional subtyping of cell lines. Moffitt Classification, per gene, per cell across basal and classical subtypes in **a)** MP2-A, **b)** MP2-B, and **c)** MP-C displaying how a sparse expression of subtype genes across thousands of cells in each sample forces an artificial classification.

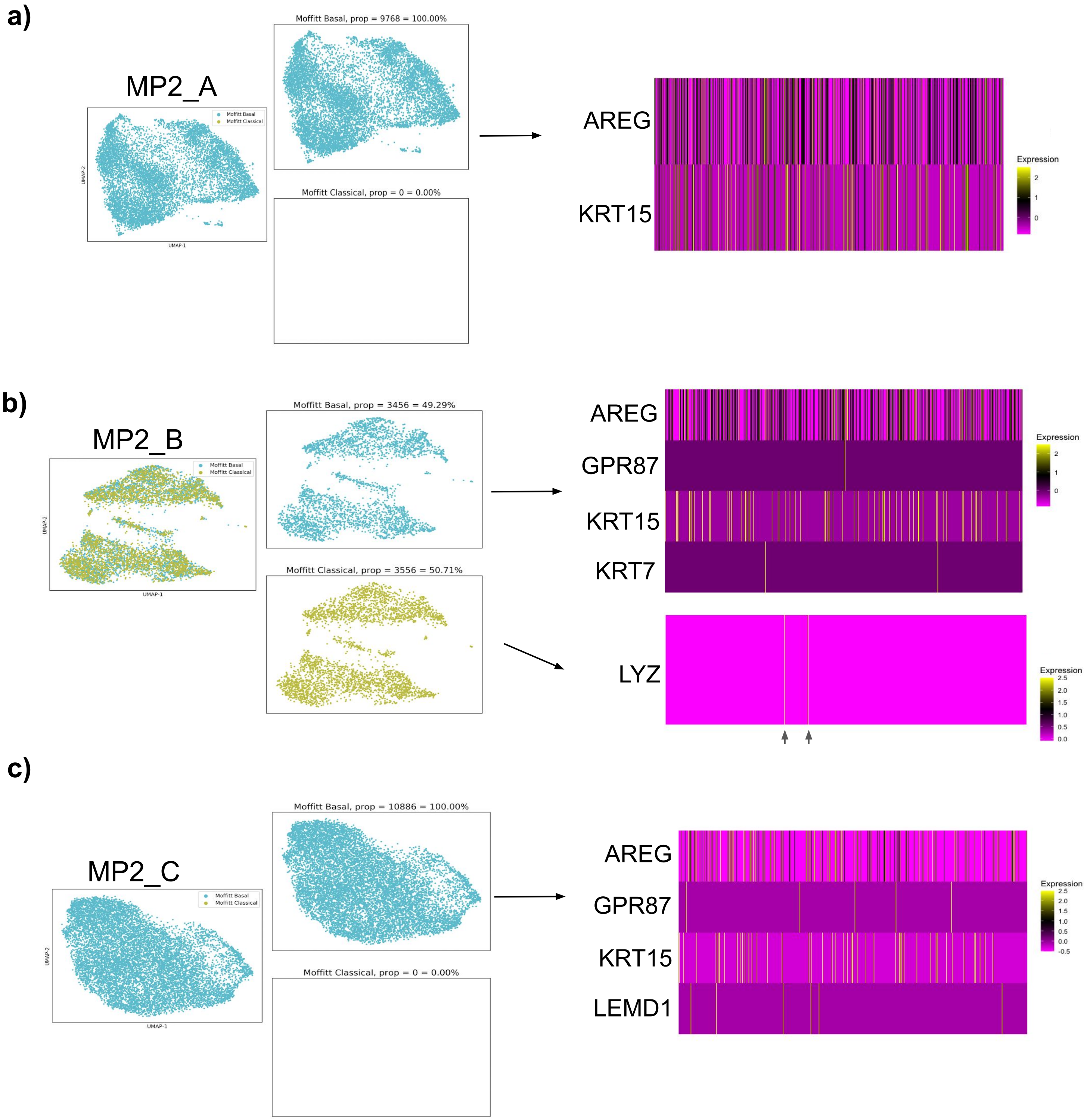

Supplementary Table 3. Distribution of PDAC scRNA cells across Collisson + Moffit PDAC subtypes. Cell Count / Percentage of total.

Cell Line	Moffitt- Basal	Moffitt- Classical	Collisson- Classical	Collisson- Exocrine	Collisson- Quasimesenchymal
MP2-A	9768 / 100%	0 / 0%	0 / 0%	0 / 0%	5420 / 55.49%
MP2-B	3456 / 49.29%	3556 / 50.71%	2408 / 34.34%	1896 / 27.04%	2708 / 38.62%
MP2-C	10886 / 100%	0 / 0%	3276 / 30.09%	3724 / 34.21%	3886 / 35.70%
BXPC3	5606 / 54.88%	4609 / 45.12%	3532 / 34.58%	2175 / 21.29%	4508 / 44.13%
HPAF-II	2367 / 51.27%	2250 / 48.73%	1777 / 38.49%	961 / 20.81%	1879 / 40.70%
HPDE	2879 / 55.41%	2317 / 44.59%	1594 / 30.68%	1479 / 2.46%	2123 / 40.86%
HPNE	6318 / 49.80%	6370 / 50.20%	518 / 4.08%	5221 / 41.15%	6949 / 54.77%
Panc1-3D	7440 / 51.03%	7139 / 48.97%	4883 / 33.49%	3537 / 24.26%	6159 / 42.25%
Panc1-2D	2891 / 53.76%	2487 / 46.24%	1671 / 31.07%	1331 / 24.75%	2376 / 44.18%

Supplementary Fig. 8) Genome-transcriptome mapping from parental Panc1 scCNVseq yields differential expression profiles between monolayer and spheroids. a) Clonealign results using the same genomic scCNV background. Clones demarcated by CNV events at chromosomes 14q and 15q. Cells per clonal group are along the x-axis, y-axis indicates CNV events used for subsetting clones. **b)** Clonealign scCNV clone correspondence to Panc1 2D scRNA data. **c)** UMAP of scCNV-derived clones mapped to corresponding scRNA-derived cells for Panc1 2D and **d)** Panc1 3D. **e)** inferCNV derived from scRNAseq of Panc1 2D and Panc1 3D, HPNE as reference. Black box depicts differential chr15 deletion, corroborating clonealign findings for scCNV-based clone designations.

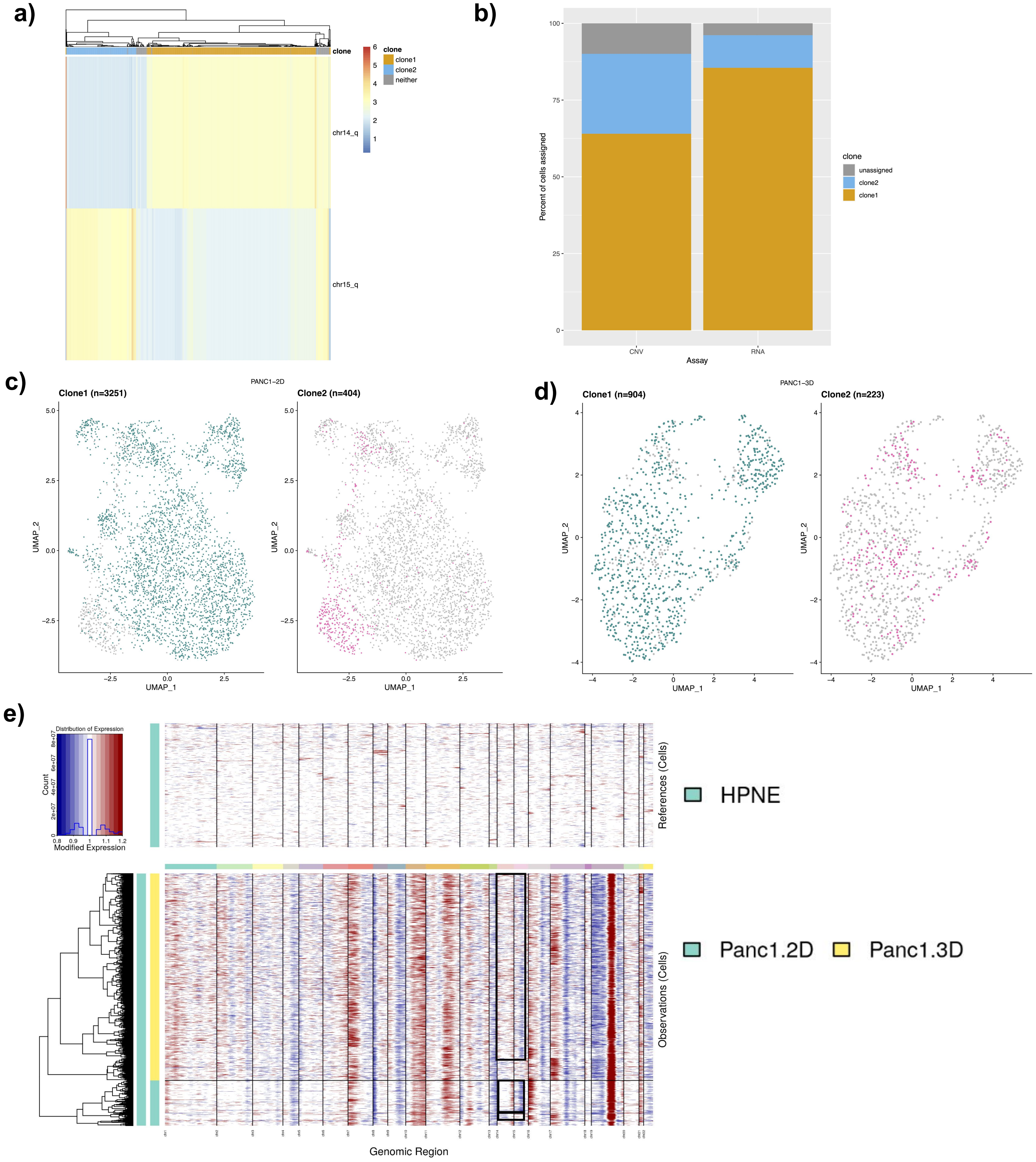

Supplementary Fig. 9) Chromatin modifications have transcriptional consequences in spheroid model. Quality-control metrics for single nuclei sequenced and analyzed for **(a)** Panc1 spheroids and **(b)** Panc1 monolayer samples. **c)** Individual analysis of Panc1 spheroid nuclei shows 3 predominating states of chromatin architecture (clusters 0-2) that correspond to unique enrichment patterns. **d)** Clusters 0 and 1 of Panc1 spheroid nuclei are enriched for SP motifs, NRF1, YY2, ZBTB33, and KLF14. **e)** Cluster 2 of Panc1 spheroid nuclei is enriched for FOS/Jun motifs. **f)** Individual analysis of Panc1 2D (monolayer) nuclei shows 2 predominating states of chromatin architecture (clusters 1, 2) that correspond to unique enrichment patterns. **g)** Cluster 1 of Panc1 monolayer nuclei is enriched for Fos/Jun motifs, ZBED1, CENPB, CLOCK, and BHLHE22. **h)** Cluster 2 of Panc1 monolayer nuclei is enriched for MYBL1, GBX1, BARX1, VENTX, T, and OTX1.

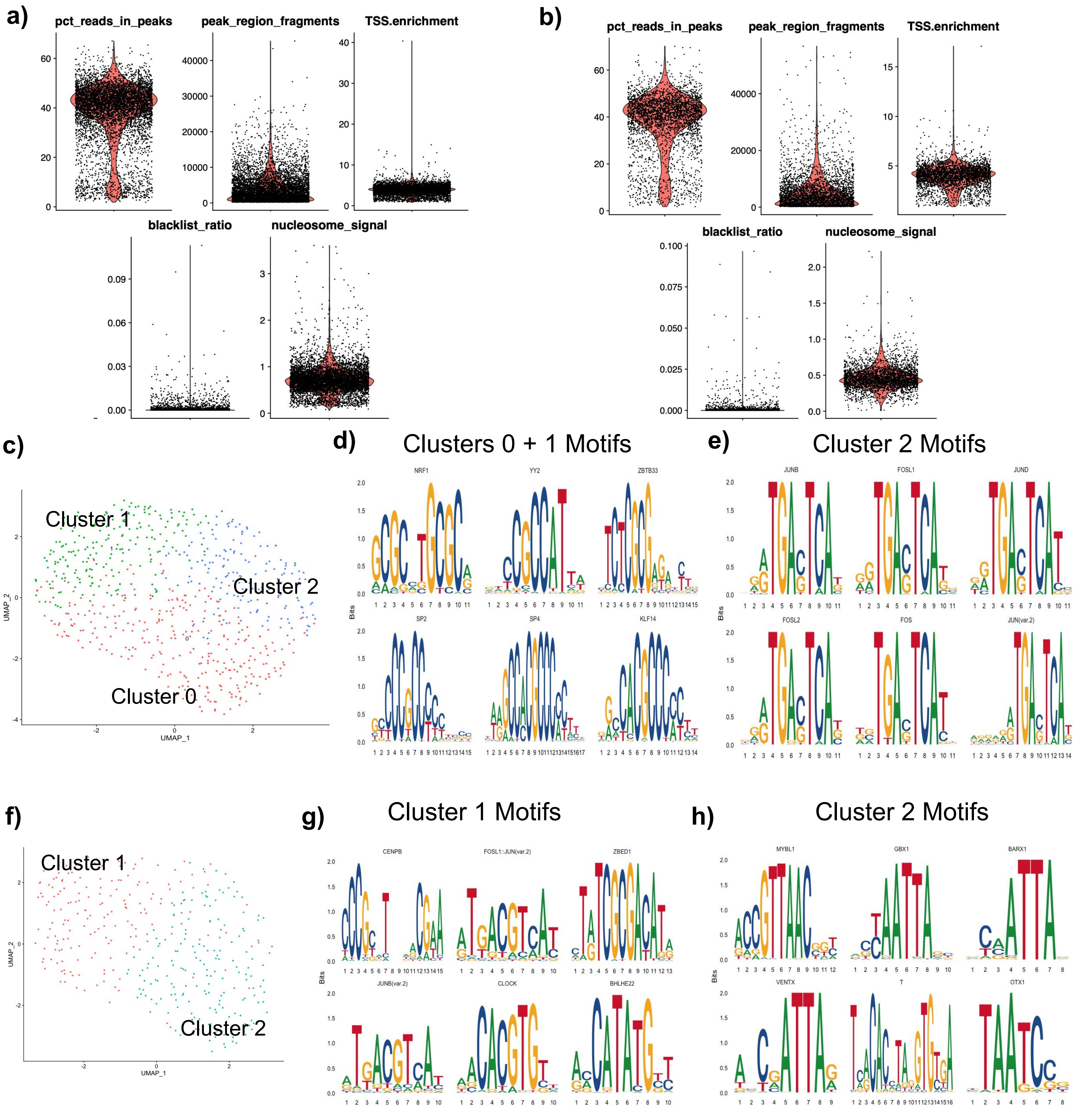

Supplementary Fig. 10) PDO evolves towards molecular subtype admixture over time. a) Heatmap annotated per cell in Early versus Late PDO culture (columns) and genes (rows) associated with Moffitt Classical and Basal molecular subtypes. PDO1 early expresses genes almost exclusively associated with the Basal Subtype, while many cells within PDO1 late express genes that are either classical or basal in nature.

a)

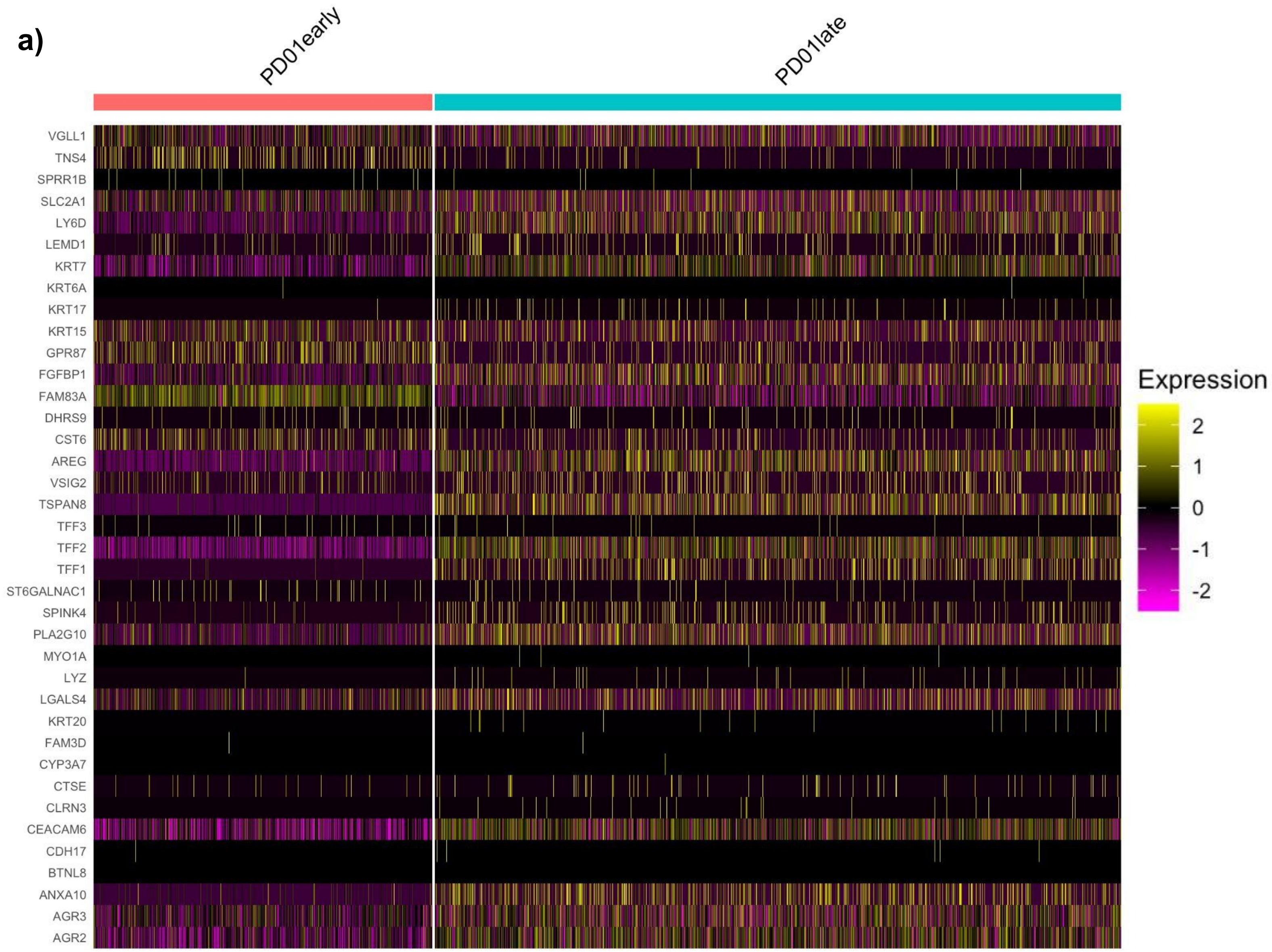

Supplement: Supplementary file 1 — Supplementary Figures and Tables [file 41467_2022_31376_MOESM1_ESM.pdf]
