# Supplementary material for: Occult polyclonality of preclinical pancreatic cancer models drives in vitro evolution
Source: Nat Commun. 2022 Jun 25;13:3652. doi: 10.1038/s41467-022-31376-3 (PMC9233687; doi:10.1038/s41467-022-31376-3)
Supplement: Supplementary file 3 — Reporting Summary [file 41467_2022_31376_MOESM3_ESM.pdf]

nature portfolio

Corresponding author(s): Maria E. Monberg

Last updated by author(s): Apr 27, 2022

Reporting Summary

Nature Portfolio wishes to improve the reproducibility of the work that we publish. This form provides structure for consistency and transparency in reporting. For further information on Nature Portfolio policies, see our Editorial Policies and the Editorial Policy Checklist.

Statistics

For all statistical analyses, confirm that the following items are present in the figure legend, table legend, main text, or Methods section.

n/a	Confirmed	
☐	☒	The exact sample size (*n*) for each experimental group/condition, given as a discrete number and unit of measurement
☐	☒	A statement on whether measurements were taken from distinct samples or whether the same sample was measured repeatedly
☐	☒	The statistical test(s) used AND whether they are one- or two-sided<br>*Only common tests should be described solely by name; describe more complex techniques in the Methods section.*
☐	☒	A description of all covariates tested
☐	☒	A description of any assumptions or corrections, such as tests of normality and adjustment for multiple comparisons
☐	☒	A full description of the statistical parameters including central tendency (e.g. means) or other basic estimates (e.g. regression coefficient) AND variation (e.g. standard deviation) or associated estimates of uncertainty (e.g. confidence intervals)
☐	☒	For null hypothesis testing, the test statistic (e.g. *F*, *t*, *r*) with confidence intervals, effect sizes, degrees of freedom and *P* value noted<br>*Give P values as exact values whenever suitable.*
☒	☐	For Bayesian analysis, information on the choice of priors and Markov chain Monte Carlo settings
☐	☒	For hierarchical and complex designs, identification of the appropriate level for tests and full reporting of outcomes
☐	☒	Estimates of effect sizes (e.g. Cohen's *d*, Pearson's *r*), indicating how they were calculated

Our web collection on statistics for biologists contains articles on many of the points above.

Software and code

Policy information about availability of computer code

Data collection

To collect raw sequencing reads for both scCNV and scRNAseq data, Cell Ranger version 2.0 was used to convert Illumina base calls (bcls) to FASTQ files. FASTQ files for scCNV data and scRNA were aligned to the reference genome GrCh38 using files provided by 10x Genomics. Cell Ranger DNA Software v1.1 was used for subsequent downstream analysis of each cell line's aligned data. For snATACseq data, Cell Ranger version 3.1.0 and cellranger-atac version 1.2.0 were used to convert Illumina bcls to fastq files and align samples to reference genome GrCh38.
For whole exome sequencing and analysis (applies only to HPDE and HPNE samples), Bcl2Fastq (2.20.0, Illumina) was used to convert raw Illumina data (bcls) into fastqs. Fastqs were then trimmed with SureCallTrimmer (v4.0.1, AGeNT v2.0, Agilent), aligned to hg19 with BWA (Burrows-Wheeler Aligner, 0.7.15-r1140, Li 2020, and barcodes were collapsed with LocatIt (v4.0.1, AGeNT v2.0, Agilent) to family size of 1. Resulting bam files were then processed by GATK (v4.1.2.0) according to best practices13, including base quality recalibration (BQSR).

Data analysis

To analyze whole exome sequencing data (for HPNE and HPDE samples only), segmentation analysis was performed as described by GATK best practices70. Briefly, we use the PBMC data, sequenced with the same platform and library to create a CNV panel of normals (PoN) to establish a baseline. We then collected read counts for HPDE/HPNE on padded (100bp) targeted regions, and then standardized and denoised using the PoN. Read counts were then standardized and denoised before making segmentation calls.

Seurat (v3.1, Butler 2018) merged analysis was used to profile the scRNA cell line data. Copy number alterations from scRNA-seq were inferred using inferCNV R package v1.0.4 (https://github.com/broadinstitute/inferCNV).
For downstream analysis of snATACseq data, Using Seurat v3 and its extension package Signac version 1.0.0 (https://github.com/timoast/signac), along with additional R packages for gene annotation (EnsDb.Hsapiens.v86) and the JASPAR 2020 Motif Database (JASPAR2020), 10x genomic output files (metadata, fragments file, fragments index, filtered peak matrices) to generate Seurat objects containing motif information, gene annotations, and genomic ranges.

For integration of scCNV-scRNAseq data on a per-sample basis, the mappable region coordinates from 10x Genomics Cell Ranger (v1.1) outputs were intersected with the gene coordinates (from Ensembl v84, same as in the CellRanger reference) to produce a gene x clone matrix. This matrix was used as input to clonealign (v2.0), which was used to assign cells in the RNA to each clone.

For all GSEA analysis described in the Main Text, pre-ranked GSEA was performed via the GSEA software version GSEA_4.0.3. Molecular Signature Databases h.all.v7.1.symbols_1.gmt (hallmark pathways) and c6.all.v7.2.symbols.gmt (oncogenic signature gene sets) were both used to align gene lists extracted from scRNA clones in Seurat.

For the PDO described in the text, trajectory inference was performed using Monocle v2.13.0

For manuscripts utilizing custom algorithms or software that are central to the research but not yet described in published literature, software must be made available to editors and reviewers. We strongly encourage code deposition in a community repository (e.g. GitHub). See the Nature Portfolio guidelines for submitting code & software for further information.

Data

Policy information about availability of data

All manuscripts must include a data availability statement. This statement should provide the following information, where applicable:

- Accession codes, unique identifiers, or web links for publicly available datasets
- A description of any restrictions on data availability
- For clinical datasets or third party data, please ensure that the statement adheres to our policy

All scRNAseq and scATACseq datasets generated and analyzed in the current study are available through Gene Expression Omnibus (GEO Accession: GSE173339). All data pertaining to scDNAseq generated and analyzed in this study are available in the European Nucleotide Archive (ENA Project: PRJEB44785).

Field-specific reporting

Please select the one below that is the best fit for your research. If you are not sure, read the appropriate sections before making your selection.

☒ Life sciences ☐ Behavioural & social sciences ☐ Ecological, evolutionary & environmental sciences

For a reference copy of the document with all sections, see nature.com/documents/nr-reporting-summary-flat.pdf

Life sciences study design

All studies must disclose on these points even when the disclosure is negative.

Sample size	Number of cells per sample for each sequencing type was determined based on targeting parameters specified by 10x Genomics protocols (CNV: Demonstrated Protocol CG000153, Rev C, scRNA 3prime: Demonstrated Protocol CG00052, Rev F, snATAC: Demonstrated Protocol CG000169, Rev D) based on viability and cell count at time of cell harvesting. Additionally, number of cells per sample were loaded into the Chromium Chip such that sequencing depth requirements for Illumina NextSeq500 would be met, also specified by protocols provided by 10x Genomics.
Data exclusions	Any cells excluded from the analysis were done so in accordance with quality control parameters to ensure to proper sequencing depth, coverage, and mitochondrial content. For scRNA-scCNV integration, clonealign-specific parameters were followed to ensure that data stringency across data types was upheld. This resulted in the exclusion of certain cell lines from this specific portion of our analyses, as described in Methods and the Main Text.
Replication	The single cell data sets presented in this article range from 1,000-12,000+ cells per sample, depending on data type (scCNV, scRNA, snATAC). As such, each sample harbors thousands of internal replicates. To minimize the technical variability between sequencing runs, samples were sequenced together, in a single sequencing batch, according to their data type, to eliminate the potential of “sequencing-induced” batch effects. Further, we collected a minimum of 3 data "types" for each sample, and used each dataset to orthogonally validate findings from the others. As such, the findings we report have been reproduced "internally", as s function of study design, for all samples and data elements.
Randomization	Does not apply. As we explain in our Main Text and Methods, it was critical to the study design that the identities and origin of each cell line and sample studied here were known to the authors. As such, no randomization was done.
Blinding	This study aimed to directly test genomic and transcriptional differences across presumptively "same" cell lines, culture conditions, and widely-used normal controls. For this reason, blinding was not applicable to study design or to the hypotheses tested here.

Reporting for specific materials, systems and methods

We require information from authors about some types of materials, experimental systems and methods used in many studies. Here, indicate whether each material, system or method listed is relevant to your study. If you are not sure if a list item applies to your research, read the appropriate section before selecting a response.

Materials & experimental systems

n/a	Involved in the study
☒	☐ Antibodies
☐	☒ Eukaryotic cell lines
☒	☐ Palaeontology and archaeology
☒	☐ Animals and other organisms
☐	☒ Human research participants
☒	☐ Clinical data
☒	☐ Dual use research of concern

Methods

n/a	Involved in the study
☒	☐ ChIP-seq
☒	☐ Flow cytometry
☒	☐ MRI-based neuroimaging

Eukaryotic cell lines

Policy information about cell lines

Cell line source(s)	hTERT-HPNE (ATCC Cat. CRL-4023, passage no. 1)<br>HPDE (Kerafast Cat. ECA001-FP, passage no. 4)<br>MP2-A cell line from the American Type Culture Collection (ATCC Cat. CRL-1420). MP2-B and MP2-C were authenticated to "match" MP2-A cells (ATCC Cat. CRL-1420) at the bulk level.<br>BXPC3 (ATCC Cat. CRL-1687, passage no. 1)<br>HPAF-II (ATCC Cat. CRL-1997, passage no. 1)<br>Panc-1 (ATCC Cat. CRL-1469, passage no. 3)
Authentication	All samples were submitted to MD Anderson core facilities for Fingerprinting and were authenticated accordingly.
Mycoplasma contamination	A volume of 3mL of media from each cell line was harvested and sent to the MD Anderson core facilities for mycoplasma testing and received "Negative" results prior to proceeding with cell harvesting and sequencing.
Commonly misidentified lines (See ICLAC register)	Not applicable, no commonly misidentified cell lines were used in this study.

Human research participants

Policy information about studies involving human research participants

Population characteristics	There is no population data for clinical research participants to describe for this work. scRNAseq data from a patient-derived organoid is presented here. This patient was female aged 55 at time of sample collection, and the primary tumor was a confirmed pancreatic adenocarcinoma at MD Anderson Cancer Center.
Recruitment	There are no recruitment biases to disclose, as this is not a study pertaining to clinical trials whatsoever, nor are we interrogating data pertaining to clinical outcomes, trial results, etc. The PDO sample analyzed in Figure 4 was collected in concordance with written consent following institutional review board approval (Lab00-396 and PA15-0014) at MD Anderson Cancer Center.
Ethics oversight	Again, all PDO sample acquisition was conducted under MDACC IRB approval for collection/research protocols (Lab00-396 and PA15-0014). The study was conducted in accordance with Good Clinical Practices concerning medical research in humans per the Declaration of Helsinki.

Note that full information on the approval of the study protocol must also be provided in the manuscript.
